# How lack of subsidized prescription drug coverage affects medication use by low-income Medicare beneficiaries

**DOI:** 10.1093/haschl/qxaf186

**Published:** 2025-10-18

**Authors:** F Ellen Loh, Bruce C Stuart, Rebecca J Hunt, Meiti Negari, Jacquelyn McRae

**Affiliations:** Department of Social, Behavioral and Administrative Sciences, Touro College of Pharmacy, 2090 Adam Clayton Powell Jr. Blvd, Room 603E, New York, NY 10027, United States; Department of Practice, Sciences and Health Outcomes Research, University of Maryland School of Pharmacy, Baltimore, MD 21201, United States; Department of Policy and Research, Pharmaceutical Research and Manufacturers of America (PhRMA), Washington, DC 20024, United States; Department of Policy and Research, Pharmaceutical Research and Manufacturers of America (PhRMA), Washington, DC 20024, United States; Department of Policy and Research, Pharmaceutical Research and Manufacturers of America (PhRMA), Washington, DC 20024, United States

**Keywords:** medicare part D, low-income subsidy, inflation reduction act

## Abstract

**Introduction:**

Beginning in 2024, the Inflation Reduction Act (IRA) extended eligibility for full Medicare Part D low-income subsidies (LIS) to all partial-subsidy recipients but did not address the issue of low participation rates for both full and partial subsidies. This study examines how lack of LIS affects medication use among Medicare beneficiaries who meet program eligibility criteria but fail to enroll and whether alternative sources of prescription coverage mitigate the lack of subsidies.

**Methods:**

This study used data from the 2019 Medicare Current Beneficiary Survey.

**Results:**

We found that LIS recipients averaged 49 prescriptions annually vs 25 for LIS-eligible non-enrollees and were 10% less likely to report cost-related nonadherence. Regression models controlling for differences between the two groups showed that LIS recipients filled 45% more prescriptions (*P* < 0.001) and that nonadherence was 1.4 times greater among those without subsidies (*P* = 0.014). Non-enrollees with unsubsidized Part D filled 39% fewer prescriptions than LIS recipients (*P* < 0.001).

**Conclusion:**

These findings provide a powerful policy endorsement of the value of Part D LIS for Medicare beneficiaries. Expanded LIS provided under provisions of the IRA together with a robust program designed to maximize LIS enrollment should lead to improved medication utilization within the eligible low-income beneficiary population.

## Introduction

The Inflation Reduction Act (IRA) of 2022 expanded subsidies under the Part D prescription drug low-income subsidy (LIS) program also known as “Extra Help” beginning in January 2024.^1^ Prior law provided “full subsidies” to beneficiaries with limited assets and incomes below 135% of the Federal Poverty Level (FPL). Those with slightly higher assets and incomes between 135% and 150% of FPL were eligible for “partial subsidies.” Full LIS subsidies cover the program premium and all cost-sharing except nominal prescription copayments for community-dwelling beneficiaries (long-term care residents on LIS pay nothing). Prior to 2024, partial subsidies provided for a reduced premium and deductible together with 15% coinsurance on all drug purchases up to the Part D initial coverage threshold, after which nominal copayments apply. New provisions under the IRA provide full subsidy benefits to all eligible recipients below 150% FPL.

However, the number of persons directly affected by this IRA provision is tiny compared with the much larger population eligible for or receiving full subsidies. It is estimated that approximately 300,000 Medicare beneficiaries received partial LIS subsidies in 2024 compared with more than 12 million receiving full benefits.^2^ To understand the true value of Part D subsidies on beneficiary medication use it is necessary to address enrollment among all persons eligible for the program regardless of subsidy status.

Historically, fewer than 40% of beneficiaries eligible for partial subsidies have enrolled.^3-5^ While enrollment rates for full LIS subsidies are higher because beneficiaries with coverage under Medicaid, Supplemental Security Income or a Medicare Savings Program are automatically enrolled for full-subsidy LIS, several million more beneficiaries not covered by these programs are estimated to be putatively eligible for full subsidies but remain unenrolled.^5^

Lack of knowledge among potential program eligibles together with complex application procedures^6-9^ are generally acknowledged as responsible for low LIS participation. In the past, limited efforts by the Social Security Administration,^10^ and a Federally funded outreach program managed by the National Council on Aging^11^ focused on helping low-income beneficiaries navigate the complexities of program enrollment. On June 12, 2023, the Department of Health and Human Services announced a new set of tools designed to identify and enroll new LIS recipients.^12^ First is a national data extract prepared by the Centers for Medicare & Medicaid Services (CMS) that identifies characteristics of beneficiaries most likely to be eligible but unenrolled. Second is a targeted outreach effort by the Administration for Community Living (ACL) to use the CMS data to “reach, screen, and enroll” new participants in the Extra Help program.

We believe that one way to encourage higher participation is by touting the impact of subsidies on prescription use and adherence. The research presented in this article is directed toward that end. Our objective is to ascertain the impact that subsidized prescription coverage has on medication use and adherence among beneficiaries otherwise eligible for partial and full low-income subsidies. We have 3 hypotheses. First is that LIS-eligible non-enrollees will fill fewer prescriptions and experience more cost-related nonadherence (CRN) than LIS recipients. Second is that other forms of prescription coverage held by LIS-eligible non-enrollees may partially mitigate the effects of lack of subsidies, but that higher cost sharing requirements associated with alternative coverage would result in lower use and higher CRN. Finally, we posit a counter-hypothesis that lower utilization and adherence rates observed among non-enrollees might arise because they are in better health with lower rates of chronic diseases commonly treated with prescription medications.

## Study data and methods

This study was considered exempt from review by the lead author's institutional review board. We followed STROBE (Strengthening the Reporting of Observational Studies in Epidemiology) reporting guidelines.

### Data source and study design

The analysis used data from the 2019 Medicare Current Beneficiary Survey (MCBS) limited data set files to test the study hypotheses. The MCBS is uniquely suited for this purpose because it contains administrative records for LIS enrollment together with detailed information on income and assets necessary to identify individuals who are putatively eligible for LIS but not enrolled. Data for 2019 were used because, at the time of the analysis, this was the last year before the COVID pandemic which could have skewed LIS participation rates.

We employed a retrospective cross-sectional design in which the study outcomes were ascertained for both LIS recipients and LIS-eligible non-enrollees over the same period of time.

### Primary outcomes and covariates

The outcomes for the primary hypothesis and the counter-hypothesis were self-reported prescription fills and cost-related nonadherence reported by MCBS respondents during 3 rounds of surveys in 2019. In addition to survey questions about medication use, respondents are asked to keep diaries of their medical care plus medication containers which are reviewed by surveyors during their in-home interviews. Cost-related nonadherence is assessed through questions asking whether, due to cost, the respondent had: (1) failed to fill a prescription, (2) skipped doses, (3) delayed filling a prescription, or (4) taken smaller doses than prescribed.

The secondary outcome–type of prescription coverage held–was ascertained only for the LIS-eligible non-enrolled group. The categories included: Part D (without subsidy), private coverage, other public coverage, or no coverage.

The study included the following covariates. Demographic characteristics included age, sex, race/ethnicity, marital status, educational attainment, metro residence, and geographic region. Economic indicators included whether respondents obtained income from work and if so, how much annual total income in relation to the FPL, and assets countable toward LIS eligibility determination (see below). Beneficiary health was assessed with a general health status question plus 10 indicator variables for specific chronic disease: diabetes, heart conditions (myocardial infarction, angina pectoris or coronary heart disease, congestive heart failure, other heart conditions), dementia (Alzheimer's or non-Alzheimer's dementia), hypertension, mental illness (depression or other mental illness), osteoporosis, cancer (non-skin), Parkinson's disease, stroke, and lung disease (emphysema, asthma, COPD).

### Identification of study subjects

The study was restricted to community-dwelling beneficiaries. Those with one or more months of LIS enrollment in 2019 were identified from administrative records and constituted the LIS recipient sample. Slightly more than 92% of these individuals were LIS enrolled for the entire year. For the remainder, enrollment duration was scattered over 11 months and was too small to meaningfully analyze. The upshot of our sample choice is that we have underestimated the full effect of LIS enrollment on medication use and adherence by approximately 4% due to the fact that these individuals were not covered by LIS for between one and 11 months during 2019.

Putatively LIS-eligible non-enrollees were identified through the following steps using survey responses obtained during the summer MCBS Income and Asset (I&A) survey segment. LIS eligibility was based on adjusted income. Our first adjustment was to subtract the LIS unearned income disregard of $20 per month from annual income. Next, among beneficiaries reporting earned income, we subtracted the LIS earned income disregard up to $65 per month plus half of any remaining earned income. Because the I&A questionnaire only quantifies earned income from the month prior to the survey round, we approximated adjusted annual earnings by multiplying monthly earned income by 12 (for those who died during 2019 we multiplied monthly earning by the number of months lived during the year). Finally, we determined whether adjusted income fell below the LIS income criteria for 2019: $16 862 (single) and $22 829 (couple) for full-subsidy LIS and $18 735 (single) and $25 365 (couple) for partial-subsidy LIS.

Next, we determined whether beneficiaries were asset-eligible for LIS based on full-subsidy asset limits of $9230 (single) and $14 600 (couple) and partial-subsidy asset limits of $14 610 (single) and $28 720 (couple). Countable assets included checking and savings account balances, certificates of deposit, mutual funds, stocks and bonds, individual retirement accounts, and the values of a business, farm or other real estate. All beneficiaries who met these criteria and were not receiving LIS were considered putatively eligible for subsidies.

### Statistical analysis

In unadjusted comparisons, we used chi-square tests to establish statistically significant differences in characteristics of LIS recipients and putatively eligible non-enrollees. We used *t*-tests to distinguish differences in rates of prescription fills and probability of CRN between the two groups. All values were weighted to be generalizable to the entire community-dwelling Medicare population.

The conditional tests of our primary hypothesis used an ordinary least squares (OLS) regression model for prescription fills and a logistic regression model for any CRN, both containing all demographic and economic variables listed above. The main independent variable of interest was a binary measure representing LIS enrollment. To challenge the primary hypothesis, we estimated additional regressions adding the general health and chronic condition variables.

We also estimated an OLS model and a logistic regression model to test the second hypotheses regarding potential mitigating effect of other prescription coverage on prescription fills and CRN. These models were restricted to the LIS-eligible non-enrolled group. The independent variables included all demographic, economic, and health variables listed above together with a set of prescription coverage variables. The strength of the second hypothesis was assessed on coefficient values for prescription fills and CRN by type of coverage maintained in relation to no prescription coverage.

### Limitations

As with all observational designs, the results from this study cannot be interpreted as causal. The study outcomes were based on self-reported data and are subject to recall bias in addition to the sampling errors common to all surveys. Another limitation is the fact that the results can only be generalized to the community-dwelling Medicare population. The comparatively small sample of putatively LIS-eligible individuals may have contributed to the lack of statistically significant differences compared with LIS recipients on some variables of interest. The small sample of individuals enrolled in LIS for less than a full year also precluded analysis. We cannot rule out the possibility of selection bias among eligible beneficiaries enrolling in LIS. The analysis did not support our counter-hypothesis that health status and chronicity explained most of the observed differences in annual prescription fills and CRN between LIS recipients and non-enrollees. However, other unobserved behavioral influences and attitudes toward medication taking^13,14^ may have contributed to the large differences we observed in prescription fills. Finally, the results are based on data from 2019, and results may differ in later years.

## Study results

### Sample characteristics

The final study sample included 2178 MCBS respondents receiving LIS subsidies and 854 respondents who were putatively estimated to be eligible for subsidies but not enrolled. The weighted samples represented 10 959 022 community-dwelling Medicare beneficiaries enrolled in LIS program and 5 275 761 Medicare beneficiaries eligible for subsidies but not enrolled. As shown in Table 1, 95.3% of all LIS recipients received full subsidies, compared with just 4.7% for partial subsidies. The overall participation rate in the subsidy was 67.5%, but participation was much higher for full-subsidy recipients (72.8%) as compared to partial-subsidy recipients (27.4%).

**Table 1.** Comparison of characteristics of LIS recipients to those putatively eligible for subsidies but not enrolled: community-dwelling medicare beneficiaries in 2019.

	Low-income subsidy enrollment status			
	LIS enrollees	Putatively eligible for Lis but not enrolled	Difference	*P*-value
Characteristic				
Unweighted *N*	2178	854	—	—
Weighted *N*	10 959 022	5 275 761	—	—
LIS eligibility status				<0.001
Full subsidy	95.3%	74.1%	21.3%	
Partial-subsidy	4.7	25.9	−21.3	
Age				<0.001
<65 disabled	41.1	18.0	23.1	
65 to 74	34.1	45.3	−11.3	
75 to 84	18.1	26.3	−8.2	
85+	6.8	10.4	−3.6	
Sex				0.296
Female	61.9	59.2	2.7	
Male	38.1	40.8	−2.7	
Self-reported race/ethnicity				<0.001
Non-Hispanic white	46.6	66.4	−19.8	
Non-Hispanic black	24.4	16.7	7.7	
Hispanic	19.4	11.7	7.7	
Other race	9.6	5.2	4.4	
Marital status				<0.001
Married	22.6	39.8	−17.1	
Widowed	20.6	26.4	−5.7	
Single	25.1	10.6	14.6	
Divorced	31.6	23.3	8.3	
Highest education level				<0.001
Less than high school	35.4	23.0	12.3	
High school grad	32.0	35.7	−3.7	
Some college	25.1	31.6	−6.4	
College grad	7.5	9.7	−2.2	
Individual/spouse had income from work				<0.001
Yes	12.6	26.3	−13.7	
No	87.4	73.7	13.7	
Household income in relation to FPL				<0.001
≤100% FPL	58.2	27.4	30.8	
100%–135% FPL	24.9	36.5	−11.6	
135%–150% FPL	4.1	20.4	−16.2	
>150% FPL^a^	12.7	15.7	−3.0	
Household Asset level				<0.001
$0	14.8	5.0	9.8	
>$0–$1000	48.6	33.5	15.1	
>$1000–$2000	20.6	23.7	−3.1	
>$2000–$6000	9.6	22.6	−13.1	
>$6000	6.5	15.2	−8.7	
Metro area residence				0.177
Metro	78.7	75.9	2.8	
Rural	21.3	24.1	−2.8	
Geographic region				0.057
Northeast	18.0	17.4	0.6	
Midwest	17.9	21.7	−3.8	
South	43.5	46.4	−3.0	
West	20.7	14.5	6.2	
General health status				<0.001
Excellent/very good	26.0	41.1	−15.1	
Good	29.8	30.9	−1.1	
Fair/poor	43.7	28.0	15.7	
Specific chronic condition				
Diabetes	41.7	33.8	7.9	<0.001
Heart conditions	30.4	31.4	−1.0	0.634
Dementia	5.0	2.8	2.2	0.008
Arthritis	28.6	22.3	6.3	0.002
Hypertension	66.1	63.0	3.1	0.206
Mental illness	44.9	29.4	15.4	<0.001
Osteoporosis	18.6	17.1	1.6	0.380
Cancer (non-skin)	15.6	17.2	−1.6	0.391
Parkinson's disease	1.4	1.2	0.2	0.692
Stroke	14.0	11.8	2.2	0.178
Lung disease	28.8	20.8	8.1	<0.001
Prescription coverage				
Part D with LIS	100.0	0.0	—	—
Part D without LIS	0.0	66.5	33.5	—
Private coverage	0.0	11.2	98.8	—
Other public coverage	0.0	7.0	93.0	—
No Rx coverage	0.0	15.4	84.6	—

Source: Authors' analysis of the 2019 Medicare Current Beneficiaries Survey (MCBS) data.

The percentage breakdowns shown in this table are based on weighted *N*'s.

Abbreviations: FPL, Federal Poverty Level; LIS, Low-income Subsidy.

^a^Some individuals may have income exceeding the LIS income threshold of 150% FPL either because of income disregards or errors in self-reported income.

Table 1 also shows differences in characteristics between LIS enrolled and LIS-eligible but non-enrolled beneficiaries. LIS recipients were significantly more likely to be disabled, <65 years old, non-white, single or divorced and non-working, with lower levels of education, income, and assets. LIS recipients had higher rates of chronic illness for most conditions assessed and had poorer general health status as compared to non-enrollees. Two-thirds of non-enrollees had Part D coverage without subsidies and just 15% had no prescription coverage.

### Unadjusted differences in prescription fills and cost-related nonadherence

Table 2 compares the annual average number of prescription fills (left-hand columns) and nonadherence rates (right-hand columns) for both groups in 2019. LIS recipients filled an annual average of 49 prescriptions, nearly double the rate (25 prescriptions) for the LIS-eligible non-enrolled group (*P* < 0.001). The differential was large for both those eligible for full subsidies (48.8 prescriptions vs 22.7 prescriptions; *P* < 0.001) and partial subsidies (55.4 prescriptions vs 30.2 prescriptions; *P* = 0.034). Annual prescription fills were consistently higher among LIS recipients across all categories of beneficiary characteristics including the 10 chronic disease variables included in the study. Among non-enrolled beneficiaries with unsubsidized Part D coverage, annual prescription fills (30.4) were triple those with no Rx coverage (10.2), but still substantially below the fills of LIS recipients with Part D (49.1) (*P* < 0.001 in each case).

**Table 2.** Comparison of annual prescription fills and rates of cost-related nonadherence by LIS recipients to those putatively eligible for subsidies but not enrolled: community-dwelling medicare beneficiaries in 2019.

	Annual prescription fills				Percent with cost-related nonadherence			
Characteristic	LIS enrollees	Putatively eligible for LIS but not enrolled	Difference	*P*-value	LIS enrollees	Putatively eligible for LIS but not enrolled	Difference	*P*-value
Overall	49.1	24.6	24.5	<0.001	22.3%	25.1%	−2.8%	0.178
Type of CRN								
Fail to fill Rx	—	—	—	—	13.2	17.7	−4.5	0.004
Skip Rx doses	—	—	—	—	6.6	8.1	−1.5	0.256
Delay filling Rx	—	—	—	—	10.6	13.8	−3.2	0.026
Take smaller doses	—	—	—	—	9.2	9.0	0.2	0.904
LIS eligibility status								
Full subsidy	48.8	22.7	26.1	<0.001	21.4	23.7	−2.3	0.320
Partial-subsidy	55.4	30.2	25.2	0.034	39.3	29.0	10.3	0.140
Age								
<65 disabled	53.3	26.8	26.6	<0.001	28.7	40.4	−11.7	0.030
65 to 74	46.3	21.0	25.3	<0.001	21.1	27.0	−6.0	0.104
75 to 84	46.8	28.6	18.3	<0.001	14.0	17.4	−3.4	0.282
85+	46.6	29.1	17.5	<0.001	11.0	9.3	1.7	0.660
Sex								
Female	53.9	25.4	28.5	<0.001	24.9	27.4	−2.5	0.415
Male	40.9	23.5	17.4	<0.001	18.0	21.7	−3.7	0.258
Self-reported race/ethnicity								
Non-Hispanic white	50.6	24.4	26.2	<0.001	22.9	25.2	−2.3	0.440
Non-Hispanic black	49.2	24.6	24.6	<0.001	23.1	30.0	−7.0	0.288
Hispanic	45.7	22.8	22.9	<0.001	17.3	18.8	−1.4	0.714
Other race	49.1	31.6	17.6	0.072	26.8	21.5	5.3	0.540
Marital status								
Married	50.4	27.6	22.8	<0.001	22.9	27.0	−4.1	0.321
Widowed	49.2	23.7	25.5	<0.001	18.7	20.0	−1.3	0.710
Single	41.0	16.5	24.5	<0.001	21.9	30.0	−8.1	0.239
Divorced	53.8	25.4	28.4	<0.001	24.3	25.2	−0.8	0.848
Highest education level								
Less than high school	49.3	29.2	20.1	<0.001	17.7	21.0	−3.3	0.298
High school grad	46.0	22.4	23.6	<0.001	21.4	20.3	1.1	0.711
Some college	51.8	25.6	26.2	<0.001	29.3	34.0	−4.7	0.297
College grad	52.2	20.1	32.2	<0.001	23.6	23.0	0.7	0.929
Individual/spouse had income from work								
Yes	39.9	21.4	18.5	0.001	21.4	23.4	−2.0	0.379
No	50.5	25.8	24.8	<0.001	28.1	29.7	−1.6	0.756
Household income in relation to FPL								
≤100% FPL	50.3	23.3	27.0	<0.001	20.5	24.5	−3.9	0.303
100%–135% FPL	46.2	22.7	23.5	<0.001	26.2	22.9	3.3	0.419
135%–150% FPL	52.4	31.7	20.7	0.040	28.4	27.4	0.9	0.893
>150% FPL	48.3	23.1	25.1	0.001	20.5	28.0	−7.4	0.221
Household asset level								
$0	49.0	20.9	28.1	<0.001	21.9	31.7	−9.8	0.249
>$0–$1000	51.5	23.4	28.1	<0.001	23.8	21.0	2.9	0.366
>$1000–$2000	45.4	23.7	21.7	<0.001	20.7	25.9	−5.3	0.172
>$2000–$6000	45.4	27.6	17.7	0.021	18.4	26.1	−7.7	0.164
>$6000	50.0	25.7	24.3	0.005	22.0	29.0	−7.0	0.386
Metro area residence								
Metro	47.6	23.1	24.4	<0.001	21.6	25.0	−3.4	0.129
Rural	54.4	29.7	24.7	<0.001	24.8	25.3	−0.5	0.927
Geographic region								
Northeast	53.3	22.9	30.5	<0.001	15.8	24.5	−8.7	0.022
Midwest	51.0	23.8	27.2	<0.001	22.3	27.3	−5.0	0.199
South	51.5	28.1	23.4	<0.001	25.8	25.3	0.5	0.899
West	36.7	15.6	21.1	<0.001	20.4	21.5	−1.1	0.839
General health status								
Excellent/very good	36.4	18.5	17.9	<0.001	11.0	19.5	−8.5	0.003
Good	45.2	25.3	20.0	<0.001	19.0	23.7	−4.7	0.233
Fair/poor	60.8	33.9	26.9	<0.001	30.7	35.8	−5.1	0.223
Specific chronic condition								
Diabetes	59.9	36.0	23.9	<0.001	25.6	30.0	−4.4	0.219
Heart conditions	63.7	36.3	27.4	<0.001	24.4	30.2	−5.7	0.184
Dementia	59.6	35.2	24.4	0.033	19.0	26.6	−7.6	0.394
Arthritis	63.9	37.3	26.5	<0.001	27.9	31.0	−3.1	0.411
Hypertension	56.6	32.6	23.9	<0.001	23.7	25.5	−1.8	0.460
Mental illness	61.7	30.9	30.8	<0.001	28.6	39.3	−10.7	0.009
Osteoporosis	64.4	33.4	31.0	<0.001	27.6	35.1	−7.6	0.192
Cancer (non-skin)	61.6	29.7	31.9	<0.0001	23.6	25.0	−1.3	0.791
Parkinson's disease	73.0	33.0	40.0	0.031	22.6	33.7	−11.1	0.562
Stroke	61.0	31.6	29.5	<0.001	20.6	28.5	−7.9	0.193
Lung disease	69.7	30.3	39.4	<0.001	27.6	39.7	−12.2	0.008
Prescription coverage								
Part D with LIS	49.1	0.0	—	—	22.3	0.0	—	—
Part D without LIS	0.0	30.4	18.7	—	0.0	26.7	4.4	—
Private coverage	0.0	23.8	25.3	—	0.0	15.7	−6.6	—
Other public coverage	0.0	5.9	43.2	—	0.0	27.8	5.5	—
No Rx coverage	0.0	10.2	38.9	—	0.0	23.6	1.3	—

Source: Authors' analysis of the 2019 Medicare Current Beneficiaries Survey (MCBS) data.

Abbreviations: CRN, cost-related nonadherence; FPL, Federal Poverty Level; LIS, Low-income Subsidy.

As shown in the right-hand columns of Table 2, a surprisingly high percentage of both groups reported CRN, albeit few of the unadjusted differences met conventional levels of statistical significance. Among LIS recipients 22.3% experienced some form of CRN compared with 25.1% for non-enrollees (*P* = 0.178). The most common form of CRN reported among non-enrollees was failure to fill prescriptions (13.2% for LIS recipients and 17.7% for non-enrollees; *P* = 0.004). LIS non-enrollees also reported higher rates of skipped doses compared with LIS enrollees (8.1% vs 6.6%; *P* = 0.256) and delayed filling (13.8% vs 10.6%; *P* = 0.026).

### Regression results

Table 3 presents results from our regression analysis of the conditional relationship between LIS status and annual prescription fills (left-hand columns) and cost-related nonadherence (right-hand columns). Model 1 in each case represents the test of our primary hypothesis that LIS subsidies generate greater demand for medications and reduce CRN, whereas Model 2 tests the counter-hypothesis that differences in health status are the main reasons for observed differences between LIS recipients and LIS-eligible non-enrollees.

**Table 3.** Regression results showing the impact of low-income subsidy enrollment status on prescription fills and cost-related nonadherence.

	Annual prescription fills				Cost-related nonadherence			
	Parameter estimate				Odds ratio			
Independent variable	Model 1	*P*-value	Model 2	*P*-value	Model 1	*P*-value	Model 2	*P*-value
LIS enrollment status								
LIS recipient	—	—	—	—	—	—	—	—
LIS-eligible but not enrolled	−23.3	<0.001	−16.9	<0.001	1.2	0.266	1.4	0.014
LIS eligibility status								
Full subsidy	—	—	—	—	—	—	—	—
Partial-subsidy	5.8	0.290	5.2	0.290	2.1	<0.001	2.0	<0.001
Age								
<65 disabled	12.0	0.001	6.7	0.034	1.7	0.002	1.4	0.090
65 to 74	—	—	—	—	—	—	—	—
75 to 84	1.8	0.476	2.4	0.336	0.6	<0.001	0.6	0.010
85+	0.9	0.772	−0.3	0.936	0.4	<0.001	0.4	0.003
Sex								
Female	—	—	—	—	—	—	—	—
Male	−9.5	<0.001	−2.5	0.214	0.6	<0.001	0.7	0.014
Self-reported race/ethnicity								
Non-Hispanic white	—	—	—	—	—	—	—	—
Non-Hispanic black	0.0	0.990	−0.9	0.771	1.2	0.436	1.2	0.375
Hispanic	0.0	1.000	−3.9	0.237	0.9	0.700	0.9	0.538
Other race	3.4	0.571	−2.9	0.598	1.4	0.191	1.4	0.223
Marital status								
Married	—	—	—	—	—	—	—	—
Widowed	−4.4	0.224	−3.8	0.253	0.9	0.428	0.9	0.391
Single	−14.6	<0.001	−5.2	0.113	0.7	0.050	0.9	0.455
Divorced	−0.6	0.884	0.3	0.945	0.8	0.185	0.8	0.186
Highest education level								
Less than high school	—	—	—	—	—	—	—	—
High school grad	−6.4	0.065	−4.9	0.117	1.0	0.793	1.0	0.892
Some college	−0.7	0.856	−4.5	0.202	1.5	0.012	1.6	0.012
College grad	3.3	0.490	4.5	0.224	1.3	0.354	1.4	0.233
Individual/spouse had income from work								
Yes	—	—	—	—	—	—	—	—
No	9.6	0.001	4.9	0.088	0.8	0.117	0.7	0.026
Household jncome in relation to the FPL								
≤100% FPL	—	—	—	—	—	—	—	—
100%–135% FPL	−2.5	0.319	−3.2	0.184	1.2	0.231	1.2	0.298
135%–150% FPL	2.8	0.612	0.1	0.980	0.9	0.760	0.9	0.699
>150% FPL	0.5	0.905	−2.3	0.492	0.8	0.177	0.7	0.182
Household asset level								
$0	−1.9	0.643	−3.7	0.322	1.1	0.577	1.1	0.629
>$0–$1000	—	—	—	—	—	—	—	—
>$1000–$2000	−3.4	0.270	−0.9	0.761	0.9	0.552	0.9	0.632
>$2000–$6000	1.2	0.760	1.5	0.650	1.0	0.865	1.0	0.856
>$6000	−1.1	0.818	1.1	0.800	1.0	0.890	1.0	0.890
Metro area residence								
Metro	—	—	—	—	—	—	—	—
Rural	5.4	0.199	3.4	0.340	1.0	0.800	1.0	0.983
Geographic region								
Northeast	—	—	—	—	—	—	—	—
Midwest	−3.1	0.490	−4.7	0.217	1.2	0.472	1.2	0.479
South	−1.6	0.651	−1.7	0.558	1.4	0.132	1.4	0.130
West	−13.7	0.000	−9.5	0.002	1.1	0.668	1.2	0.443
General health status								
Excellent/very good			—	—			—	—
Good			1.8	0.424			1.4	0.068
Fair/poor			6.7	0.015			2.1	<0.0001
Specific chronic condition								
Diabetes			10.9	0.001			10.6	0.005
Heart conditions			9.9	0.001			1.2	0.143
Dementia			1.1	0.820			1.1	0.482
Arthritis			8.1	0.005			1.0	0.983
Hypertension			13.0	<0.001			1.3	0.093
Mental illness			12.0	<0.001			1.1	0.598
Osteoporosis			7.6	0.009			1.4	0.019
Cancer (non-skin)			5.2	0.098			1.3	0.125
Parkinson's disease			7.8	0.542			0.9	0.707
Stroke			2.7	0.400			1.1	0.932
Lung disease			12.2	0.001			0.8	0.256
R-square			0.136	0.308				

Source: Authors' analysis of the 2019 Medicare Current Beneficiaries Survey (MCBS) data.

Abbreviations: FPL, Federal Poverty Level; LIS, Low-income Subsidy.

Controlling for demographic and financial resource variables (Model 1) explained little of the difference in annual prescription fills between non-enrollees and LIS recipients (−23.3 fills; *P* < 0.001) compared with −24.5 fills (*P* < 0.001) in the unadjusted comparisons. The only variables to reach statistical significance in this model were age <65, single marital status, had income from work, and resided in the West. Adding the health variables (Model 2) reduced the difference in fills between the non-enrollees and LIS recipients by just over a quarter to −16.9, a difference that remained highly significant (*P* < 0.001). All the health variables except for cancer, Parkinson's disease, and stroke were significant.

Table 3 also presents the rate of CRN after controlling for potential confounders. After controlling for demographic and financial resource variables (Model 1), the rate of CRN was 1.2 times higher among non-enrollees than LIS recipients (*P* = 0.266), and the rate of CRN was 1.4 times higher among non-enrollees as compared to LIS recipients after also controlling for health variables (ie, Model 2) (*P* = 0.014). Non-enrollees putatively eligible for partial subsidies were twice as likely to experience CRN compared with those putatively eligible for full subsidies in both Models 1 and 2 (*P* < 0.001). Other significant coefficients in these models included age, sex, fair/poor health, and diabetes. Prescription fills were slightly higher among beneficiaries eligible for partial vs full LIS (5.8 and 5.2 more fills in the two models, respectively) but neither result was statistically significant. However, rates of reported CRN were significantly higher among those eligible for partial subsidies (OR = 2.1; *P* < 0.001 and OR = 2.0; *P* < 0.001).

Table 4 presents regression results showing the impact of alternative prescription coverage on fills and CRN among putatively LIS-eligible non-enrollees. Compared with beneficiaries with no prescription coverage, enrollment in Part D without subsides raised annual prescription fills by 15.6 (*P* < 0.001) compared with 7.5 for those with private coverage (*P* = 0.061). The impact of other public coverage (−3.3; *P* = 0.439) appears to be an anomaly of small sample size. None of the alternative types of prescription coverage had a significant impact on CRN. It is also noteworthy that when we restricted the analysis to non-enrollees, we found no significant differences in drug use or adherence between those eligible for partial and full LIS.

**Table 4.** Regression results assessing the impact of alternative drug coverage on prescription fills and cost-related nonadherence among those putatively LIS-eligible but not enrolled.

	Annual prescription fills		Cost-related nonadherence	
Independent variable	Parameter estimate	*P*-value	Odds ratio	*P*-value
Prescription coverage				
Part D without LIS	15.6	<0.001	1.1	0.838
Private coverage	7.5	0.061	0.4	0.131
Other public coverage	−3.3	0.439	1.2	0.701
No Rx coverage	—	—	—	—
LIS eligibility status				
Full subsidy	—	—	—	—
Partial-Subsidy	−0.7	0.906	1.6	0.224
Age				
<65 disabled	5.2	0.327	1.7	0.157
65 to 74	—	—	—	—
75 to 84	4.6	0.152	0.6	0.071
85+	3.7	0.280	0.3	0.014
Sex				
Female	—	—	—	—
Male	2.9	0.249	0.7	0.195
Self-reported race/ethnicity				
Non-Hispanic white	—	—	—	—
Non-Hispanic black	−2.0	0.690	1.6	0.152
Hispanic	−0.7	0.889	0.9	0.680
Other race	−2.8	0.570	0.7	0.572
Marital status				
Married	—	—	—	—
Widowed	0.1	0.981	0.8	0.544
Single	0.0	0.996	0.9	0.692
Divorced	4.8	0.154	0.6	0.061
Highest education level				
Less than high school	—	—	—	—
High school grad	0.0	0.989	0.9	0.826
Some college	−0.7	0.862	1.8	0.037
College grad	1.0	0.871	1.4	0.458
Individual/spouse had income from work				
Yes	—	—	—	—
No	−1.6	0.750	0.6	0.181
Household income in relation to FPL				
≤100% FPL	—	—	—	—
100%–135% FPL	−1.5	0.526	0.8	0.534
135%–150% FPL	5.2	0.391	0.7	0.441
>150% FPL	−4.9	0.401	0.6	0.280
Household asset level				
$0	−4.4	0.243	1.9	0.340
>$0–$1000	—	—	—	—
>$1000–$2000	−0.4	0.907	1.4	0.284
>$2000–$6000	2.7	0.481	1.5	0.268
>$6000	2.6	0.586	1.5	0.283
Metro area residence				
Metro	—	—	—	—
Rural	4.1	0.138	1.0	0.924
Geographic region				
Northeast	—	—	—	—
Midwest	−0.8	0.829	0.8	0.579
South	4.1	0.187	0.7	0.365
West	−0.7	0.861	0.8	0.571
General health status				
Excellent/very good	—	—	—	—
Good	5.4	0.040	1.0	0.917
Fair/poor	8.2	0.016	1.3	0.353
Specific chronic condition				
Diabetes	8.5	0.003	1.1	0.619
Heart conditions	9.1	0.003	1.3	0.377
Dementia	5.5	0.524	1.7	0.429
Arthritis	7.2	0.076	1.3	0.326
Hypertension	10.9	<0.001	1.0	0.958
Mental illness	2.9	0.373	1.8	0.028
Osteoporosis	6.3	0.013	1.6	0.137
Cancer (non-skin)	2.4	0.500	1.0	0.966
Parkinson's disease	−5.4	0.487	1.7	0.632
Stroke	1.4	0.706	0.9	0.682
Lung disease	−0.7	0.872	2.3	0.002

Source: Authors' analysis of the 2019 Medicare Current Beneficiaries Survey (MCBS) data.

Abbreviations: FPL, Federal Poverty Level; LIS, Low-income Subsidy.

## Discussion

To our knowledge, this paper is the first study to document the impact of LIS on prescription drug use. Our study outcomes captured two complementary aspects of drug use: (1) prescription fills, which we hypothesized would be higher among LIS recipients compared with non-enrollees; and (2) cost-related nonadherence, which we expected to be higher among non-enrollees. We hypothesized that the driver in both cases is the substantially reduced out-of-pocket costs paid by subsidy recipients, although this was not independently assessed.

There is extensive literature on how insurance and cost sharing influences drug use. A recent systematic review of the literature on the association of prescription coverage and medication use found that for the U.S. population as a whole, a 10% increase in the out-of-pocket cost of medications was associated with an average reduction in use of between 2% and 5%.^15^ Studies focused on aged populations have found similar levels of price responsiveness among elderly individuals facing higher out-of-pocket drug costs.^16,17^ Studies of populations with poor health and with high levels of chronicity have reported declines in medication use of between 3% and 5% per 10% increase in out-of-pocked cost.^15-17^

Prior research has also reported that relatively large numbers of low-income Medicare beneficiaries and LIS recipients face CRN issues.^18^ A study by Schluterman and McCormick found that 23.3% of beneficiaries with annual incomes below $25 000 faced CRN in 2016 compared with 15.8% with incomes above that level.^19^ An earlier study by Wei et al. found that 22% of LIS recipients reported CRN in 2007.^13^

As with prior research on LIS participation rates,^6-9^ we found that substantial numbers of putatively eligible beneficiaries were not enrolled. The participation rate was particularly low among those eligible for partial subsidies (27.4%) compared with 72.8% of those eligible for full subsidies; the latter due primarily to auto-enrollment of beneficiaries receiving Medicaid, SSI, or a Medicare Savings Program. However, overall, many more beneficiaries were eligible for full subsidies in 2019 as compared to partial subsidies. The actual number of those putatively eligible but not enrolled in the full-subsidy LIS was 3.9 million persons in 2019, which is nearly 3 times the number of those eligible but not enrolled for partial subsidies (1.4 million persons).

These findings have important policy implications. The IRA's LIS provisions focus only on LIS eligibility and do not address the problem of low participation rates. It is possible that raising subsidy levels in 2024 will draw in additional enrollees among those with incomes between 135% and 150% of FPL. But there is no inducement in the law to attract the much larger numbers of putatively eligible beneficiaries with incomes below 135% of FPL.

One tactic that might improve participation rates is for the government and low-income advocacy groups to communicate more broadly the benefits and emphasize the benefits that LIS subsidies provide to recipients in terms of improved access to needed medications and better medication adherence. This could easily be incorporated in the new outreach program being implemented by the ACL and CMS.

Finally, in light of the attention given the issue of Medicaid cuts under the recently enacted One Big Beautiful Bill Act (OBBBA), it is worth considering how the OBBBA will affect LIS enrollment rates. The Congressional Budget Office estimates that the legislation will result in 6.2 million fewer Medicaid recipients by 2035,^20^ but those affected are between the ages of 19 and 64. There is nothing in the law that directly affects the aged and disabled who are currently eligible for LIS. Nonetheless, higher state Medicaid costs in the future could result in unintended consequences for LIS enrollment in coming years.

## Conclusions

The results of this study provide compelling evidence of the value of the Part D LIS program to low-income Medicare beneficiaries. In multivariable regression analyses, LIS recipients were found to fill significantly more prescriptions and experience significantly lower levels of cost-related nonadherence compared with putatively eligible beneficiaries who were not enrolled in LIS. The two groups did differ in terms of health status and chronicity, but together these variables explained just over a quarter of the observed differences in fills and CRN. Whether the increased LIS benefits promised under the IRA will induce more eligible low-income Medicare beneficiaries to enroll in 2024 and later years remains to be seen. Success may well depend on whether eligible beneficiaries are sufficiently well informed about the value of LIS enrollment to voluntarily enroll.

## Supplementary Material

qxaf186_Supplementary_Data
